# Activation Phenotype of *Mycobacterium tuberculosis*-Specific CD4^+^ T Cells Promoting the Discrimination Between Active Tuberculosis and Latent Tuberculosis Infection

**DOI:** 10.3389/fimmu.2021.721013

**Published:** 2021-08-26

**Authors:** Ying Luo, Ying Xue, Liyan Mao, Qun Lin, Guoxing Tang, Huijuan Song, Wei Liu, Shutao Tong, Hongyan Hou, Min Huang, Renren Ouyang, Feng Wang, Ziyong Sun

**Affiliations:** ^1^Department of Laboratory Medicine, Tongji Hospital, Tongji Medical College, Huazhong University of Science and Technology, Wuhan, China; ^2^Department of Immunology, School of Basic Medicine, Tongji Medical College, Huazhong University of Science and Technology, Wuhan, China

**Keywords:** activation phenotype, HLA-DR, *Mycobacterium tuberculosis*, discrimination, active tuberculosis, latent tuberculosis infection

## Abstract

**Background:**

Rapid and effective discrimination between active tuberculosis (ATB) and latent tuberculosis infection (LTBI) remains a challenge. There is an urgent need for developing practical and affordable approaches targeting this issue.

**Methods:**

Participants with ATB and LTBI were recruited at Tongji Hospital (Qiaokou cohort) and Sino-French New City Hospital (Caidian cohort) based on positive T-SPOT results from June 2020 to January 2021. The expression of activation markers including HLA-DR, CD38, CD69, and CD25 was examined on *Mycobacterium tuberculosis* (MTB)-specific CD4^+^ T cells defined by IFN-γ, TNF-α, and IL-2 expression upon MTB antigen stimulation.

**Results:**

A total of 90 (40 ATB and 50 LTBI) and another 64 (29 ATB and 35 LTBI) subjects were recruited from the Qiaokou cohort and Caidian cohort, respectively. The expression patterns of Th1 cytokines including IFN-γ, TNF-α, and IL-2 upon MTB antigen stimulation could not differentiate ATB patients from LTBI individuals well. However, both HLA-DR and CD38 on MTB-specific cells showed discriminatory value in distinguishing between ATB patients and LTBI individuals. As for developing a single candidate biomarker, HLA-DR had the advantage over CD38. Moreover, HLA-DR on TNF-α^+^ or IL-2^+^ cells had superiority over that on IFN-γ^+^ cells in differentiating ATB patients from LTBI individuals. Besides, HLA-DR on MTB-specific cells defined by multiple cytokine co-expression had a higher ability to discriminate patients with ATB from LTBI individuals than that of MTB-specific cells defined by one kind of cytokine expression. Specially, HLA-DR on TNF-α^+^IL-2^+^ cells produced an AUC of 0.901 (95% CI, 0.833–0.969), with a sensitivity of 93.75% (95% CI, 79.85–98.27%) and specificity of 72.97% (95% CI, 57.02–84.60%) as a threshold of 44% was used. Furthermore, the performance of HLA-DR on TNF-α^+^IL-2^+^ cells for differential diagnosis was obtained with validation cohort data: 90.91% (95% CI, 72.19–97.47%) sensitivity and 68.97% (95% CI, 50.77–82.73%) specificity.

**Conclusions:**

We demonstrated that HLA-DR on MTB-specific cells was a potentially useful biomarker for accurate discrimination between ATB and LTBI.

## Introduction

Tuberculosis (TB), caused by *Mycobacterium tuberculosis* (MTB) infection, remains a lethal infectious disease that needs to be paid more attention globally (1–3). It was reported that there were still an estimated 10 million cases and nearly 1.4 million deaths of the disease in 2019 (4). The one main hurdle in controlling TB is the difficulty in differentiating active TB (ATB) from latent TB infection (LTBI) (5–8). Delayed ATB identification would enhance TB transmission and hamper the global End TB strategy (9, 10). Therefore, the development of tools for discrimination between ATB and LTBI would be a priority in the global effort to combat this disease (11, 12).

Pathogen detections including smear microscopy and mycobacterial culture were applied widely in the TB diagnosis field. However, the drawbacks of these conventional tests included limited sensitivity and length of time consumed (13, 14). Although a rapid diagnosis has been enhanced by various molecular techniques such as GeneXpert MTB/RIF and GeneXpert MTB/RIF Ultra (15–20), these tests also showed unsatisfactory utility, especially for paucibacillary cases (21–24). Interferon gamma release assays, including T-SPOT.TB (T-SPOT) and QuantiFERON-TB Gold In-Tube (QFT-GIT), were widely used for identifying MTB infection (25–28). Nevertheless, these two tests were not useful in distinguishing ATB from LTBI (29–31). Moreover, a variety of host biomarkers identified by omics including transcriptome (32–34), proteome (35, 36), and metabolome (37–39) have been proposed as potential biomarkers for diagnosing MTB infection. Howbeit, few studies validated their actual benefit in various areas with different demographic, ethnic settings, as well as TB prevalence. Besides, high operating costs and infrastructural requirements hinder their use in resource-constrained settings. Hence, there is an imperative need to seek an approach with good performance based on existing technology platforms.

Previous studies showed that MTB-specific T-cell activation might associate with MTB antigen load (40, 41). Several publications had denoted the potential of antigen-specific HLA-DR and CD38 in TB diagnosis (41, 42). Notwithstanding, further validation for these investigations, especially in different populations, is needed to get the most promising biomarkers a step closer to clinical translation. Meanwhile, T-cell activation could reflect by multiple markers including HLA-DR, CD38, CD69, and CD25. HLA-DR is usually highly expressed on the surface of T cells in advanced stages of activation as one kind of human class II major histocompatibility complex antigen (43–45). CD38 is a type II transmembrane glycoprotein that is extensively expressed on cells of hematopoietic and non-hematopoietic lineage (46). It is downregulated in resting memory cells and elevates in activated cells (45, 47). CD69 acts as a costimulatory molecule for T-cell activation and proliferation (48). It is one of the early markers upregulated after T-cell activation (45, 49–51). CD25, as α chain of IL-2 receptor, plays a key role in responsiveness to IL-2, enabling T lymphocyte activation and further IL-2 production (45, 52). Different activation biomarkers may have inconsistent performance in the diagnosis of TB. Besides, CD27 acts as a differentiation marker expressed on T cells as a member of the TNF receptor family (53). It was reported that the decreased expression of this marker denoted a conversion of T cells towards effector memory phenotype (54). Several previous studies have found that CD27 expression on MTB-specific cells decreased in ATB patients (40, 55, 56), suggesting its potential in TB diagnostics. Moreover, the value of the combination of various activation and differentiation biomarkers for TB diagnostics has not been adequately elaborated. Therefore, the issue is needed to be clarified. Meanwhile, MTB-specific T cells could represent by multiple cytokine secretion profiles including IFN-γ, TNF-α, and IL-2 upon MTB antigen stimulation. Thus, there is urgent need to identify appropriate combination for TB diagnosis with optimal performance. The present study evaluated and validated the potential utility of various activation and differentiation markers on different MTB-specific cells for TB diagnosis.

## Methods

### Study Design

The present study was carried out between June 2020 and January 2021. Participants were recruited at Tongji Hospital (Qiaokou cohort, the largest tertiary hospital in central China with 5500 beds) and Sino-French New City Hospital (Caidian cohort, a branch hospital of Tongji Hospital with 1600 beds), respectively. Subjects in two cohorts were selected based on positive T-SPOT results. On the basis of clinical and laboratory assessments, participants were classified as patients with ATB and individuals with LTBI. ATB patients were diagnosed by positive mycobacterial culture and/or GeneXpert MTB/RIF with supportive symptoms and radiological findings of ATB. LTBI individuals were identified by positive T-SPOT result in the absence of ATB evidence. Exclusion criteria for the study included (1) having received anti-TB therapy for more than 2 weeks and (2) being younger than 17 years old. This study was reviewed and approved by the committee of Tongji Hospital, Tongji Medical College, Huazhong University of Science and Technology.

### T-SPOT Assay

Heparin-anticoagulated blood samples were collected for performing T-SPOT assay (Oxford Immunotec, Oxford, UK). The operation was conducted in accordance with manufacturer’s instruction. Briefly, peripheral blood mononuclear cells (PBMCs) were separated by Ficoll-Hypaque gradient centrifugation. Then, the isolated PBMCs (2.5 × 10^5^) were added to 96-well plates precoated with antibody against IFN-γ. There were four wells each participant: medium (negative control), early secreted antigenic target 6 (ESAT-6), culture filtrate protein 10 (CFP-10), and phytohemagglutinin (positive control). Cells were incubated for 16–20 h at 37°C with 5% CO_2_ and developed using anti-IFN-γ antibody conjugate with substrate to detect the presence of IFN-γ secreted cells. Spot-forming cells were counted with an automated enzyme-linked immunospot reader (CTL Analyzers, Cleveland, OH, USA). The test result was regarded positive if ESAT-6 and/or CFP-10 spot number minus negative control spot number ≥ 6. The test result was regarded negative if both ESAT-6 spot number minus negative control spot number and CFP-10 spot number minus negative control spot number ≤ 5. Results were considered undetermined if the spot number in phytohemagglutinin well was <20 or spot number in the medium well was >10.

### Detection of Markers on MTB-Specific CD4^+^ Cells

**Figure 1:**
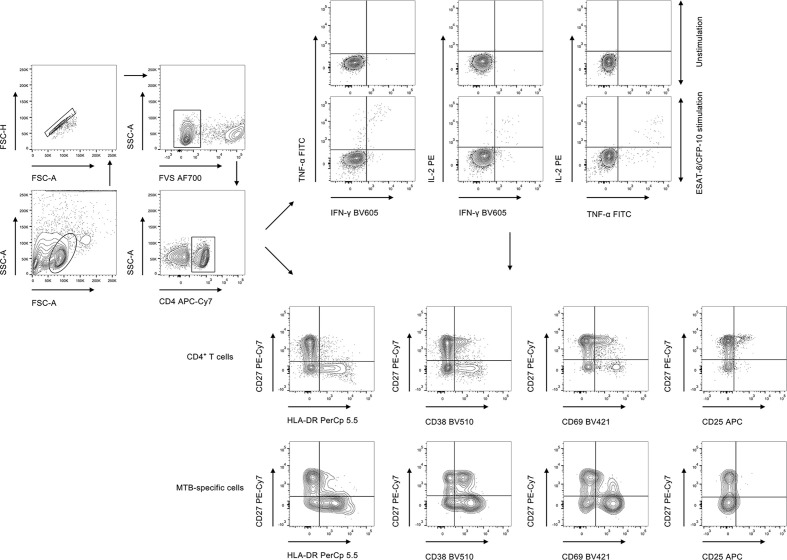
The gating strategies for cytokine expression and activation phenotype in the current study. FVS, fixable viability stain; MTB, *Mycobacterium tuberculosis*.

PBMCs were stimulated with peptide ESAT-6 (2 μg/ml) and CFP-10 (2 μg/ml) for 18 h at 37°C with 5% CO_2_. Briefly, PBMCs were counted and 1 × 10^6^ cells were added to the well. Brefeldin A was added to the mixture 6 h before staining of the cells. Post incubation, PBMCs were first stained with Fixable Viability Stain 700 (BD Pharmingen) to discriminate live from dead cells, followed by appropriate surface marker staining. Cell surface staining was performed on PBMCs using the following anti-human monoclonal antibodies: anti-CD4-APC-Cy7, anti-HLA-DR-PerCp 5.5, anti-CD38-BV510, anti-CD69-BV421, anti-CD25-APC, and anti-CD27-PE-Cy7. For intracellular staining, the cells were fixed and permeabilized with Fixation and Permeabilization Buffer (BD Biosciences). Intracellular cytokine staining was conducted using the following anti-human monoclonal antibodies: anti-IFN-γ-BV605, anti-TNF-α-FITC, and anti-IL-2-PE. The staining was performed at 4°C, and the cells were stained for 30 min. The gating strategy was shown in **Figure 1**. The information of used antibodies was presented in **Supplementary Table S1**. Isotype controls with irrelevant specificities were included as negative controls. After washing, the pellets were resuspended in 200 μl staining buffer and analyzed with FACSCanto II flow cytometer (BD Biosciences, San Jose, CA). The flow data were analyzed using Flowjo software version 10.6.2 (TreeStar, Ashland, OR).

The background in the unstimulated tube was subtracted when reporting the percentage of cytokine-producing cells in the stimulated tube. MTB-specific cells were determined by cytokine production including IFN-γ, TNF-α, and IL-2. Responders were defined as individuals with relative counts of cytokine-producing CD4^+^ T cells (more than 15 events were recorded) in MTB antigen-stimulated condition that were significantly higher than the unstimulated control (a fold change of more than 3). The 15-event cut-off for phenotypic analysis was applied to each cytokine combination. Phenotype analysis for MTB-specific CD4^+^ T cells was only performed on responders.

### Statistical Analysis

Continuous variables were described using median (interquartile range) or means ± standards deviation (SD). Categorical variables were expressed as number (%). Comparison between various groups was performed using Mann-Whitney *U* test for continuous variables, and Chi-square test or Fisher’s exact test for categorical variables. A two-side test with *P* < 0.05 was considered statistically significant.

The diagnostic performance of various biomarkers was assessed by receiver operator characteristics (ROC) curve analysis. Area under the curve (AUC), sensitivity, specificity, positive predictive value (PPV), negative predictive value (NPV), positive likelihood ratio (PLR), negative likelihood ratio (NLR), and accuracy, together with their 95% confidence intervals (CI), were determined. The comparison between various ROC AUCs was performed by using z test with the procedure of Delong et al. (57). Data analysis was performed using GraphPad Prism version 8 (GraphPad Software, San Diego, CA, USA), R 4.0.2 program (R Core Team), SPSS version 25.0 (SPSS, Inc., Chicago, IL, USA), and MedCalc version 11.6 (MedCalc, Mariakerke, Belgium).

## Results

### Demographic and Clinical Characteristics of Study Participants

Out of 90 subjects from Qiaokou cohort, 40 were ATB patients and the other 50 were LTBI individuals. Among 64 cases from the Caidian cohort, 29 were diagnosed as ATB and 35 were diagnosed as LTBI. Participants had a mean age of around 60 years and more than half were males. There was no significant difference between ATB patients and LTBI individuals in distribution of age and gender. An overview of the clinical and demographic characteristics of recruited participants was presented in **Table 1**.

**Table 1:** Demographic and clinical characteristics of study population.

Variables	Qiaokou cohort (training set)		P*	Caidian cohort (validation set)		P*	*P* ^†^
	ATB (n = 40)	LTBI (n = 50)		ATB (n = 29)	LTBI (n = 35)		
Age, years	50.8 ± 18.2	48.9 ± 16.7	0.529	52.0 ± 17.0	53.1 ± 15.7	0.981	0.351
Sex, male, %	25 (62.5%)	31 (62.0%)	0.961	17 (58.6%)	21 (60.0%)	0.911	0.721
Underlying condition or illness							
HIV infection	1 (2.5%)	0 (0.0%)	0.261	1 (3.5%)	0 (0.0%)	0.268	0.807
Diabetes mellitus	7 (17.5%)	7 (14.0%)	0.649	5 (17.2%)	3 (8.6%)	0.296	0.593
Solid tumor	5 (12.5%)	4 (8.0%)	0.48	3 (10.3%)	4 (11.4%)	0.89	0.851
Hematological malignancy	3 (7.5%)	1 (2.0%)	0.208	2 (6.9%)	1 (2.9%)	0.447	0.943
Liver cirrhosis	2 (5.0%)	1 (2.0%)	0.431	2 (6.9%)	3 (8.6%)	0.804	0.217
End-stage renal disease	5 (12.5%)	3 (6.0%)	0.282	5 (17.2%)	3 (8.6%)	0.296	0.469
Organ transplantation	2 (5.0%)	0 (0.0%)	0.11	1 (3.5%)	1 (2.9%)	0.892	0.729
Immunosuppressive condition^‡^	7 (17.5%)	7 (14.0%)	0.649	4 (13.8%)	3 (8.6%)	0.505	0.411
Positive culture for MTB	30 (75.0%)	N/A	N/A	25 (86.2%)	N/A	N/A	N/A
Positive GeneXpert MTB/RIF	31 (77.5%)	N/A	N/A	22 (75.9%)	N/A	N/A	N/A

ATB, active tuberculosis; LTBI, latent tuberculosis infection; MTB, Mycobacterium tuberculosis; N/A, not applicable. *Comparisons were performed between ATB and LTBI groups using Mann-Whitney U test, Chi-square test, or Fisher’s exact test. ^†^Comparisons were performed between Qiaokou and Caidian cohorts using Mann-Whitney U test, Chi-square test, or Fisher’s exact test. ^‡^Patients who underwent chemotherapy or took immunosuppressants within 3 months. Data were presented as means ± SD or numbers (percentages).

### Cytokine Expression Patterns Upon MTB Antigen Stimulation for Distinguishing ATB From LTBI

**Figure 2:**
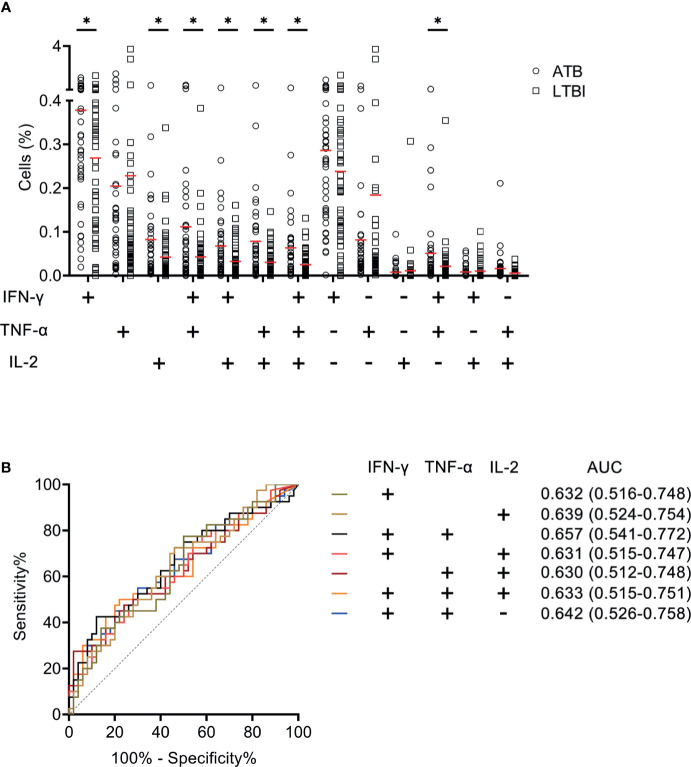
The performance of various cytokine expression pattern upon MTB antigen stimulation in distinguishing ATB patients from LTBI individuals in Qiaokou cohort. **(A)** Aligned dot plots showing the results of the expression pattern of IFN-γ, TNF-α, and IL-2 upon MTB antigen stimulation in ATB patients and LTBI individuals. Horizontal lines indicate the medians. **(B)** ROC analysis showing the performance of various cytokine expression pattern in discriminating ATB patients from LTBI individuals. *P < 0.05 (Mann-Whitney *U* test). MTB, *Mycobacterium tuberculosis*; ATB, active tuberculosis; LTBI, latent tuberculosis infection; AUC, area under the curve.

We measured the expression of Th1 cytokines including IFN-γ, TNF-α, and IL-2 secreted by CD4^+^ T cells upon MTB antigen stimulation and analyzed the value of expression profile for distinguishing ATB patients from LTBI individuals. It was observed that statistical difference existed in some co-expression patterns between patients with ATB and LTBI individuals (**Figure 2A**). When we assessed the ability of cytokine secretion patterns to diagnose ATB disease using ROC curve analysis, it was found that the AUCs produced by all candidates were lower than 0.7 (**Figure 2B**).

### Activation Markers on CD4^+^ T Cells for Discriminating ATB From LTBI

**Figure 3:**
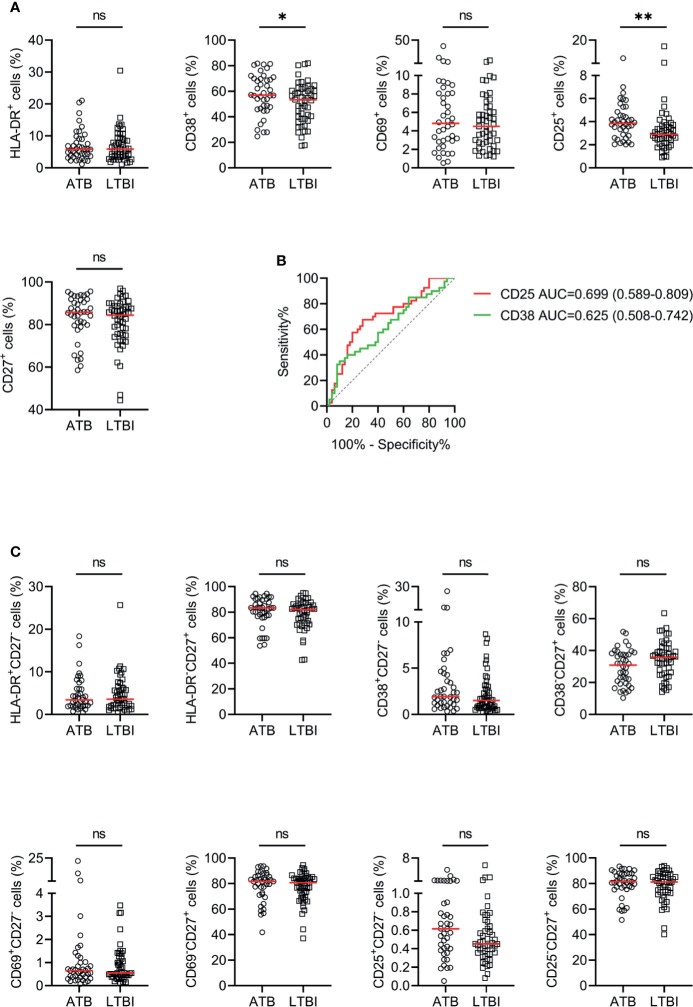
The performance of various markers on CD4^+^ T cells in distinguishing ATB patients from LTBI individuals in Qiaokou cohort. **(A)** Scatter dot plots showing the results of the expression of HLA-DR, CD38, CD69, CD25, and CD27 on CD4^+^ T cells in ATB patients and LTBI individuals. Horizontal lines indicate the medians. **(B)** ROC analysis showing the performance of the expression of CD38 and CD25 on CD4^+^ T cells in discriminating ATB patients from LTBI individuals. **(C)** Scatter dot plots showing the results of the proportions of HLA-DR^+^CD27^-^ cells, HLA-DR^-^CD27^+^ cells, CD38^+^CD27^-^ cells, CD38^-^CD27^+^ cells, CD25^+^CD27^-^ cells, and CD25^-^CD27^+^ cells of CD4^+^ T cells in ATB patients and LTBI individuals. Horizontal lines indicate the medians. *P < 0.05, **P < 0.01, ns, no significance (Mann-Whitney *U* test). MTB, *Mycobacterium tuberculosis*; ATB, active tuberculosis; LTBI, latent tuberculosis infection; AUC, area under the curve.

The utility of activation markers including HLA-DR, CD38, CD69, and CD25 on CD4^+^ T cells for discriminating ATB patients from LTBI individuals was evaluated. The expression of CD38 and CD25 in ATB patients was significantly higher than that in LTBI individuals, while no statistical difference was observed in HLA-DR and CD69 expression between these two groups (**Figure 3A**). ROC curve analysis showed that the AUCs were 0.699 (95% CI, 0.589–0.809) for CD25 and 0.625 (95% CI, 0.508–0.742) for CD38 (**Figure 3B**). When combining with CD27, no improved performance was obtained for the purpose of distinguishing ATB patients from LTBI individuals (**Figure 3C**).

### Activation Markers on MTB-Specific Cells for Differentiating ATB From LTBI

**Figure 4:**
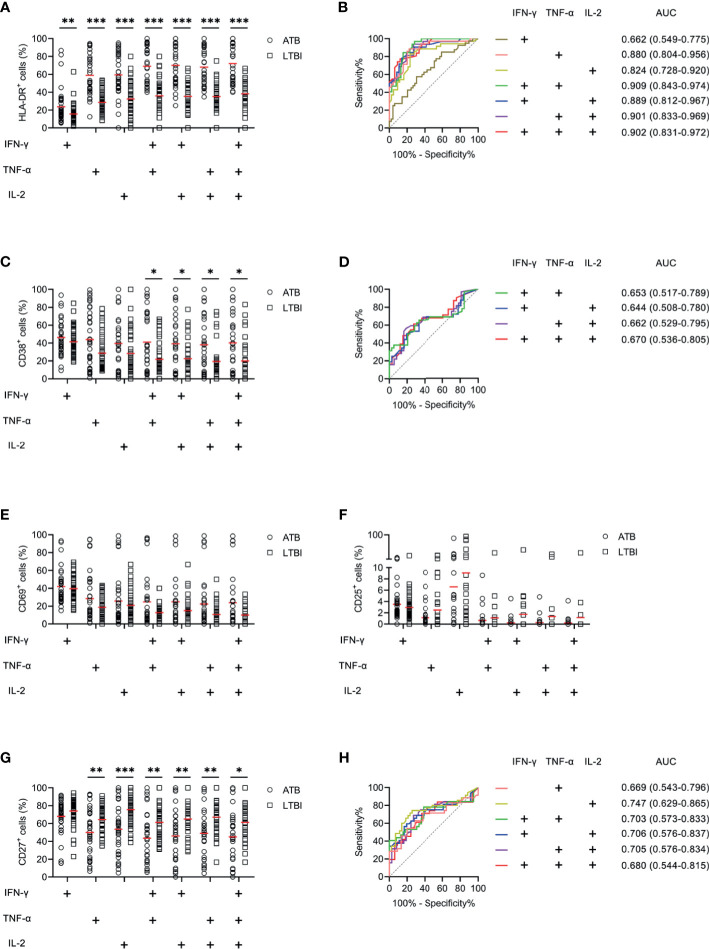
The performance of activation markers on MTB-specific CD4^+^ T cells in distinguishing ATB patients from LTBI individuals in Qiaokou cohort. **(A)** Aligned dot plots showing the results of HLA-DR expression on MTB-specific cells in ATB patients and LTBI individuals. Horizontal lines indicate the medians. **(B)** ROC analysis showing the performance of HLA-DR expression on MTB-specific cells in discriminating ATB patients from LTBI individuals. **(C)** Aligned dot plots showing the results of CD38 expression on MTB-specific cells in ATB patients and LTBI individuals. Horizontal lines indicate the medians. **(D)** ROC analysis showing the performance of CD38 expression on MTB-specific cells in discriminating ATB patients from LTBI individuals. **(E)** Aligned dot plots showing the results of CD69 expression on MTB-specific cells in ATB patients and LTBI individuals. Horizontal lines indicate the medians. **(F)** Aligned dot plots showing the results of CD25 expression on MTB-specific cells in ATB patients and LTBI individuals. Horizontal lines indicate the medians. **(G)** Aligned dot plots showing the results of CD27 expression on MTB-specific cells in ATB patients and LTBI individuals. Horizontal lines indicate the medians. **(H)** ROC analysis showing the performance of CD27 expression on MTB-specific cells in discriminating ATB patients from LTBI individuals. *P < 0.05, **P < 0.01, ***P < 0.001 (Mann-Whitney *U* test). MTB, *Mycobacterium tuberculosis*; ATB, active tuberculosis; LTBI, latent tuberculosis infection; AUC, area under the curve.

We examined the expression levels of activation markers on MTB-specific cells determined by Th1 cytokine expression. Obvious difference was found in HLA-DR and CD38 expression on MTB-specific cells between ATB patients and LTBI individuals, while no significant difference was observed in CD69 and CD25 expression between these two groups (**Figure 4**). ATB patients showed significantly higher expression of HLA-DR and CD38, especially on polyfunctional MTB-specific cells, compared to LTBI individuals (**Figures 4A, C**). Specially, when a threshold was set as 44%, HLA-DR on TNF-α^+^IL-2^+^ cells was able to discriminate ATB patients from LTBI individuals with a sensitivity of 93.75% (95% CI, 79.85–98.27%) and specificity of 72.97% (95% CI, 57.02–84.60%) (**Table 2** and **Figure 4B**). The comparison between AUCs showed that the performance of HLA-DR on TNF‐α^+^ cells was superior to that on IFN‐γ^+^ cells (Z test, *P* < 0.05). Moreover, HLA‐DR on IFN‐γ^+^TNF‐α^+^, IFN‐γ^+^IL-2^+^, and TNF‐α^+^IL-2^+^ cells had superiority over that on IFN‐γ^+^ cells in differentiating ATB from LTBI (Z test, *P* < 0.05). Meanwhile, it was observable that CD27 expression on MTB-specific cells was significantly lower in ATB patients to that in LTBI individuals (**Figures 4G, H**). The value of combination of activation markers and CD27 was also analyzed for differential diagnosis purpose. However, there was no added benefit observed (**Supplementary Figure S1**).

**Table 2:** The performance of HLA-DR on MTB-specific cells for distinguishing between ATB and LTBI in Qiaokou cohort.

Variables	Cutoff value	AUC (95% CI)	Sensitivity (95% CI)	Specificity (95% CI)	PPV (95% CI)	NPV (95% CI)	PLR (95% CI)	NLR (95% CI)	Accuracy
HLA-DR on IFN-γ^+^ cells (%)	17	0.662 (0.549–0.775)	57.50% (42.19–71.49%)	65.31% (51.31–77.08%)	57.50% (42.19–71.49%)	65.31% (51.31–77.08%)	1.66 (1.04–2.65)	0.65 (0.43–0.98)	61.80%
HLA-DR on TNF-α^+^ cells (%)	38	0.880 (0.804–0.956)	85.71% (70.63–93.74%)	77.78% (63.73–87.46%)	75.00% (59.81–85.81%)	87.50% (73.89–94.54%)	3.86 (2.2–6.77)	0.18 (0.08–0.42)	81.25%
HLA-DR on IL-2^+^ cells (%)	43	0.824 (0.728–0.920)	82.86% (67.32–91.90%)	74.42% (59.76–85.07%)	72.50% (57.17–83.89%)	84.21% (69.58–92.56%)	3.24 (1.9–5.51)	0.23 (0.11–0.49)	78.21%
HLA-DR on IFN-γ^+^TNF-α^+^ cells (%)	48	0.909 (0.843–0.974)	84.38% (68.25–93.14%)	82.05% (67.33–91.02%)	79.41% (63.20–89.65%)	86.49% (72.02–94.09%)	4.7 (2.36–9.35)	0.19 (0.08–0.43)	83.10%
HLA-DR on IFN-γ^+^IL-2^+^ cells (%)	51	0.889 (0.812–0.967)	75.00% (57.89–86.75%)	86.11% (71.34–93.92%)	82.76% (65.45–92.40%)	79.49% (64.47–89.22%)	5.40 (2.34–12.48)	0.29 (0.16–0.54)	80.88%
HLA-DR on TNF-α^+^IL-2^+^ cells (%)	44	0.901 (0.833–0.969)	93.75% (79.85–98.27%)	72.97% (57.02–84.60%)	75.00% (59.81–85.81%)	93.10% (78.04–98.09%)	3.47 (2.03–5.93)	0.09 (0.02–0.33)	82.61%
HLA-DR on IFN-γ^+^TNF-α^+^IL-2^+^ cells (%)	51	0.902 (0.831–0.972)	77.42% (60.19–88.61%)	84.85% (69.08–93.35%)	82.76% (65.45–92.40%)	80.00% (64.11–89.96%)	5.11 (2.23–11.71)	0.27 (0.14–0.52)	81.25%

MTB, Mycobacterium tuberculosis; ATB, active tuberculosis; LTBI, latent tuberculosis infection; AUC, area under the curve; PPV, positive predictive value; NPV, negative predictive value; PLR, positive likelihood ratio; NLR, negative likelihood ratio; CI, confidence interval.

### Validation of Activation Markers on MTB-Specific Cells for Differential Diagnosis Between ATB and LTBI

**Figure 5:**
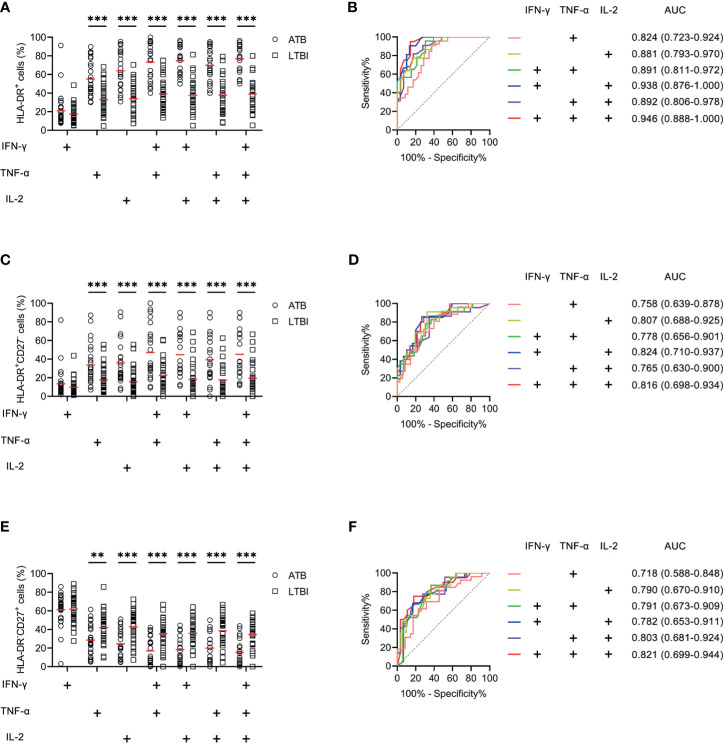
The performance of HLA-DR on MTB-specific cells in distinguishing ATB patients from LTBI individuals in Caidian cohort. **(A)** Aligned dot plots showing HLA-DR expression on MTB-specific cells in ATB patients and LTBI individuals. Horizontal lines indicate the medians. **(B)** ROC analysis showing the performance of HLA-DR expression on MTB-specific cells in discriminating ATB patients from LTBI individuals. **(C)** Aligned dot plots showing the proportions of HLA-DR^+^CD27^-^ cells of MTB-specific cells in ATB patients and LTBI individuals. Horizontal lines indicate the medians. **(D)** ROC analysis showing the performance of the proportion of HLA-DR^+^CD27^-^ cells of MTB-specific cells in discriminating ATB patients from LTBI individuals. **(E)** Aligned dot plots showing the proportions of HLA-DR^-^CD27^+^ cells of MTB-specific cells in ATB patients and LTBI individuals. Horizontal lines indicate the medians. **(F)** ROC analysis showing the performance of the proportion of HLA-DR^-^CD27^+^ cells of MTB-specific cells in discriminating ATB patients from LTBI individuals. **P < 0.01, ***P < 0.001 (Mann-Whitney *U* test). MTB, *Mycobacterium tuberculosis*; ATB, active tuberculosis; LTBI, latent tuberculosis infection; AUC, area under the curve.

Another blinded set (Caidian cohort) was enrolled to validate the performance of biomarkers discovered in Qiaokou cohort. Excellent performance of HLA-DR on polyfunctional MTB-specific cells was found for the differential diagnosis between ATB patients and LTBI individuals. With the threshold of 48%, the sensitivity and specificity of HLA-DR on IFN-γ^+^TNF-α^+^ cells were 82.61% (95% CI, 62.86–93.02%) and 69.70% (95% CI, 52.66–82.63%), respectively (**Table 3**, **Figures 5A, B**). Using a cut-off value of 51%, HLA-DR on IFN-γ^+^IL-2^+^ cells was able to discriminate between ATB patients and LTBI individuals with a specificity, sensitivity, and AUC of 83.33% (95% CI, 66.44–92.67%), 90.48% (95% CI, 71.09–97.35%), and 0.938 (95% CI, 0.876–1.000), respectively (**Table 3** and **Figures 5A, B**). When a cut-off value of 44% was used, a sensitivity of 90.91% (95% CI, 72.19–97.47%) and a specificity of 68.97% (95% CI, 50.77–82.73%) were observed in HLA-DR on TNF-α^+^IL-2^+^ cells (**Table 3** and **Figures 5A, B**). Furthermore, if using the cut-off value of 51% obtained from Qiaokou cohort, the sensitivity and specificity of HLA-DR on IFN-γ^+^TNF-α^+^IL-2^+^ cells were 95.00% (95% CI, 76.39–99.11%) and 82.14% (95% CI, 64.41–92.12%), respectively (**Table 3** and **Figures 5A, B**). Meanwhile, we also analyzed the potential value of the combination of HLA-DR and CD27 for TB diagnostic issue. Consistent with Qiaokou cohort, no obvious improvement was observed after combining CD27 (**Figures 5C–F**). **Table 3** summarized diagnostic performance of HLA-DR on MTB-specific cells when applied to the validation cohort.

**Table 3:** The performance of HLA-DR on MTB-specific cells for distinguishing between ATB and LTBI in Caidian cohort.

Variables	Cutoff value	AUC (95% CI)	Sensitivity (95% CI)	Specificity (95% CI)	PPV (95% CI)	NPV (95% CI)	PLR (95% CI)	NLR (95% CI)	Accuracy
HLA-DR on IFN-γ^+^ cells (%)	17	0.510 (0.364–0.657)	39.29% (23.56–57.59%)	60.00% (43.57–74.45%)	44.00% (26.66–62.93%)	55.26% (39.71–69.85%)	0.98 (0.53–1.81)	1.01 (0.68–1.51)	50.79%
HLA-DR on TNF-α^+^ cells (%)	38	0.824 (0.723–0.924)	76.92% (57.95–88.97%)	65.71% (49.15–79.17%)	62.50% (45.25–77.07%)	79.31% (61.61–90.16%)	2.24 (1.35–3.72)	0.35 (0.17–0.74)	70.49%
HLA-DR on IL-2^+^ cells (%)	43	0.881 (0.793–0.970)	81.82% (61.48–92.70%)	70.97% (53.41–83.91%)	66.67% (47.82–81.36%)	84.62% (66.47–93.85%)	2.82 (1.57–5.06)	0.26 (0.1–0.64)	75.47%
HLA-DR on IFN-γ^+^TNF-α^+^ cells (%)	48	0.891 (0.811–0.972)	82.61% (62.86–93.02%)	69.70% (52.66–82.63%)	65.52% (47.35–80.06%)	85.19% (67.52–94.09%)	2.73 (1.57–4.73)	0.25 (0.1–0.63)	75.00%
HLA-DR on IFN-γ^+^IL-2^+^ cells (%)	51	0.938 (0.876–1.000)	90.48% (71.09–97.35%)	83.33% (66.44–92.67%)	79.17% (59.53–90.76%)	92.59% (76.63–97.95%)	5.43 (2.41–12.23)	0.11 (0.03–0.43)	86.27%
HLA-DR on TNF-α^+^IL-2^+^ cells (%)	44	0.892 (0.806–0.978)	90.91% (72.19–97.47%)	68.97% (50.77–82.73%)	68.97% (50.77–82.73%)	90.91% (72.19–97.47%)	2.93 (1.68–5.12)	0.13 (0.03–0.51)	78.43%
HLA-DR on IFN-γ^+^TNF-α^+^IL-2^+^ cells (%)	51	0.946 (0.888–1.000)	95.00% (76.39–99.11%)	82.14% (64.41–92.12%)	79.17% (59.53–90.76%)	95.83% (79.76–99.26%)	5.32 (2.39–11.85)	0.06 (0.01–0.41)	87.50%

MTB, Mycobacterium tuberculosis; ATB, active tuberculosis; LTBI, latent tuberculosis infection; AUC, area under the curve; PPV, positive predictive value; NPV, negative predictive value; PLR, positive likelihood ratio; NLR, negative likelihood ratio; CI, confidence interval.

## Discussion

The lack of efficacious diagnostic tools poses a major challenge to control TB efforts (58, 59). Although many advances have been achieved, especially in omics field (60–62), there were some practical limitations for their clinical application, including expensive laboratory facilities and sophisticated operating procedures. Meanwhile, immunodiagnostics has received considerable attention as an alternative for discrimination of MTB infection status in recent years (63–70). Nevertheless, the identified biomarkers including proteins and cytokines in serum or plasma for diagnostic aim may not be specific for TB due to the influence brought by other immune related diseases such as infection and autoimmune diseases (71–77). Thus, an intensified search for suitable host-specific biomarkers targeting TB diagnostic purpose was urgently needed (78).

With the emergence of flow cytometry as a prominent advancement, many researchers detected makers on immune cell surface or intracellular cytokines for diagnosing infectious diseases (79–81). Some previous works showed that the immune phenotype profile was associated with MTB infection status (82). Howbeit, these evaluations might only denote the global response of the host and could not meticulously reflect the host-specific immune response to the disease. Thus, the utility of these methods is susceptible to body immunity, and their performance varies greatly in different populations, making it difficult to meet clinical diagnostic need.

Our study focused on MTB-specific cells by gating cells with Th1 cytokine secretion upon MTB antigen stimulation. It is a very small cell subset that can best reflect the host’s immune response to MTB infection. We firstly analyzed the cytokine production patterns in ATB patients and LTBI individuals. However, not as reported by previous study (83), no obvious difference was observed. Next, we simultaneously investigated the value of four activation biomarkers including HLA-DR, CD38, CD69, and CD25 on MTB-specific CD4^+^ T cells, for differentiating ATB patients from LTBI individuals. After ROC curve analysis, two biomarkers of activation (HLA-DR and CD38) showed discriminatory roles. Among them, HLA-DR was the better promising biomarker. Then, we compared the difference of HLA-DR on various MTB-specific cells. Interestingly, we found that the performance of HLA-DR on different MTB-specific cells defined by different cytokine combinations was inconsistent. HLA-DR on TNF-α^+^ or IL-2^+^ cells was remarkably superior to that on IFN-γ^+^ cells in distinguishing ATB patients from LTBI individuals. Besides, HLA-DR on polyfunctional MTB-specific cells showed a higher capability than that on MTB-specific cells defined by one cytokine secretion. These data signified the heterogeneity of MTB-specific cells, and the selection of cell subset determined the diagnostic performance of specific biomarkers. Meanwhile, an opposite trend to HLA-DR was observed in CD27 on MTB-specific cells in ATB patients and LTBI individuals. Thus, we tried to improve the efficacy for discrimination by the combination of HLA-DR with CD27. Notwithstanding, no increased or even decreased performance was obtained. Therefore, independent HLA-DR on MTB-specific cells, rather than combination with others, is more likely to be recommended as a diagnostic biomarker.

Three points should be noted: first, it was observed that HLA-DR on IFN-γ^+^ cells had relatively poor performance in distinguishing ATB from LTBI when comparing to that of TNF-α^+^ or IL-2^+^ cells. We found that the percentages of IFN-γ^+^ cells in unstimulated tubes ranged from 0.02% to 0.1%, while the percentages of TNF-α^+^ cells and IL-2^+^ cells in unstimulated tubes ranged from 0.01% to 0.04%. The background value for IFN-γ was relatively higher than that for TNF-α and IL-2. Thus, some of the IFN-γ^+^ cells in stimulated tubes were not MTB-specific cells. It may be one reason for the poor performance of HLA-DR on IFN-γ^+^ cells in differentiating ATB from LTBI. Second, the variation of HLA-DR expression in the LTBI group might be due to the different infection status. Some individuals in the LTBI group have been infected for a long time, while the others were infected with MTB recently. As indicated by previous study, some subjects with recent MTB infection would also have high HLA-DR expression on MTB-specific cells (84). Thus, another approach that could be combined with markers in the present study to improve diagnostic specificity should be developed in the future. Third, given that the increasing number of MTB antigen has been identified in recent years and the response patterns of PBMCs varied upon different antigen stimulation (85–87), the investigation targeting optimal antigen selection is needed in the future.

Several limitations should be mentioned. First, although there were two centers in the current study, the number of recruited partakers in each center was circumscribed. These biomarkers should be further validated in larger cohorts. Second, although some diseases including diabetes and tumors were involved in underlying condition of the enrolled patients in this study, further investigation is required to elucidate the influence of other diseases such as COVID-19 on the performance of these biomarkers. Finally, given the fact that the biomarkers detected in the present study were on MTB-specific cells, HLA-DR would be useless when applying to cases with few MTB-specific cells, such as T-SPOT-negative individuals (88, 89). Hence, more reasonable methods for this population should be developed in the future.

In conclusion, our study demonstrated that HLA-DR on MTB-specific cells has robust diagnostic potential for discrimination between ATB and LTBI. Notably, the detection of biomarkers discovered in the present study is amenable to the existing platforms.

## Data Availability Statement

The original contributions presented in the study are included in the article/**Supplementary Material**. Further inquiries can be directed to the corresponding authors.

## Ethics Statement

The studies involving human participants were reviewed and approved by the committee of Tongji hospital, Tongji Medical College, Huazhong University of Science and Technology. The patients/participants provided their written informed consent to participate in this study.

## Author Contributions

YL designed the study. LM, QL, and GT set up the clinical cohorts at the respective hospitals. HS, LW, ST, HH, MH, and RO included patients and collected data. YL performed the main experiment. YL analyzed and interpreted the data. YL and YX did the statistical analysis. YL draft the manuscript. YL, FW, and ZS contributed to the revision of manuscript. All authors contributed to the article and approved the submitted version.

## Funding

This work was funded by Graduate Innovation Fund of Huazhong University of Science and Technology (grant number 2021yjsCXCY088) and Special Foundation for National Science and Technology Basic Research Program of China (grant number 2019FY101206).

## Conflict of Interest

The authors declare that the research was conducted in the absence of any commercial or financial relationships that could be construed as a potential conflict of interest.

## Publisher’s Note

All claims expressed in this article are solely those of the authors and do not necessarily represent those of their affiliated organizations, or those of the publisher, the editors and the reviewers. Any product that may be evaluated in this article, or claim that may be made by its manufacturer, is not guaranteed or endorsed by the publisher.

## Supplementary Material

Supplementary Figure 1 The performance of the combination of activation markers and CD27 on MTB-specific cells in distinguishing ATB patients from LTBI individuals in Qiaokou cohort. **(A)** Aligned dot plots showing the proportions of HLA-DR^+^CD27^-^ and HLA-DR^-^CD27^+^ cells of MTB-specific cells in ATB patients and LTBI individuals. Horizontal lines indicate the medians. **(B)** ROC analysis showing the performance of the proportions of HLA-DR^+^CD27^-^ and HLA-DR^-^CD27^+^ cells of MTB-specific cells in discriminating ATB patients from LTBI individuals. **(C)** Aligned dot plots showing the proportions of CD38^+^CD27^-^ and CD38^-^CD27^+^ cells of MTB-specific cells in ATB patients and LTBI individuals. Horizontal lines indicate the medians. **(D)** ROC analysis showing the performance of the proportions of CD38^+^CD27^-^ and CD38^-^CD27^+^ cells of MTB-specific cells in discriminating ATB patients from LTBI individuals. **(E)** Aligned dot plots showing the proportions of CD69^+^CD27^-^ and CD69^-^CD27^+^ cells of MTB-specific cells in ATB patients and LTBI individuals. Horizontal lines indicate the medians. **(F)** ROC analysis showing the performance of the proportions of CD69^+^CD27^-^ and CD69^-^CD27^+^ cells of MTB-specific cells in discriminating ATB patients from LTBI individuals. **(G)** Aligned dot plots showing the proportions of CD25^+^CD27^-^ and CD25^-^CD27^+^ cells of MTB-specific cells in ATB patients and LTBI individuals. Horizontal lines indicate the medians. **(H)** ROC analysis showing the performance of the proportions of CD25^+^CD27^-^ and CD25^-^CD27^+^ cells of MTB-specific cells in discriminating ATB patients from LTBI individuals. **P* < 0.05, ***P* < 0.01, ****P* < 0.001 (Mann-Whitney *U* test). MTB, *Mycobacterium tuberculosis*; ATB, active tuberculosis; LTBI, latent tuberculosis infection; AUC, area under the curve.
