# Supplementary figures and images for: Activation Phenotype of Mycobacterium tuberculosis-Specific CD4+ T Cells Promoting the Discrimination Between Active Tuberculosis and Latent Tuberculosis Infection
Source: Front Immunol. 2021 Aug 26;12:721013. doi: 10.3389/fimmu.2021.721013 (PMC8426432; doi:10.3389/fimmu.2021.721013)

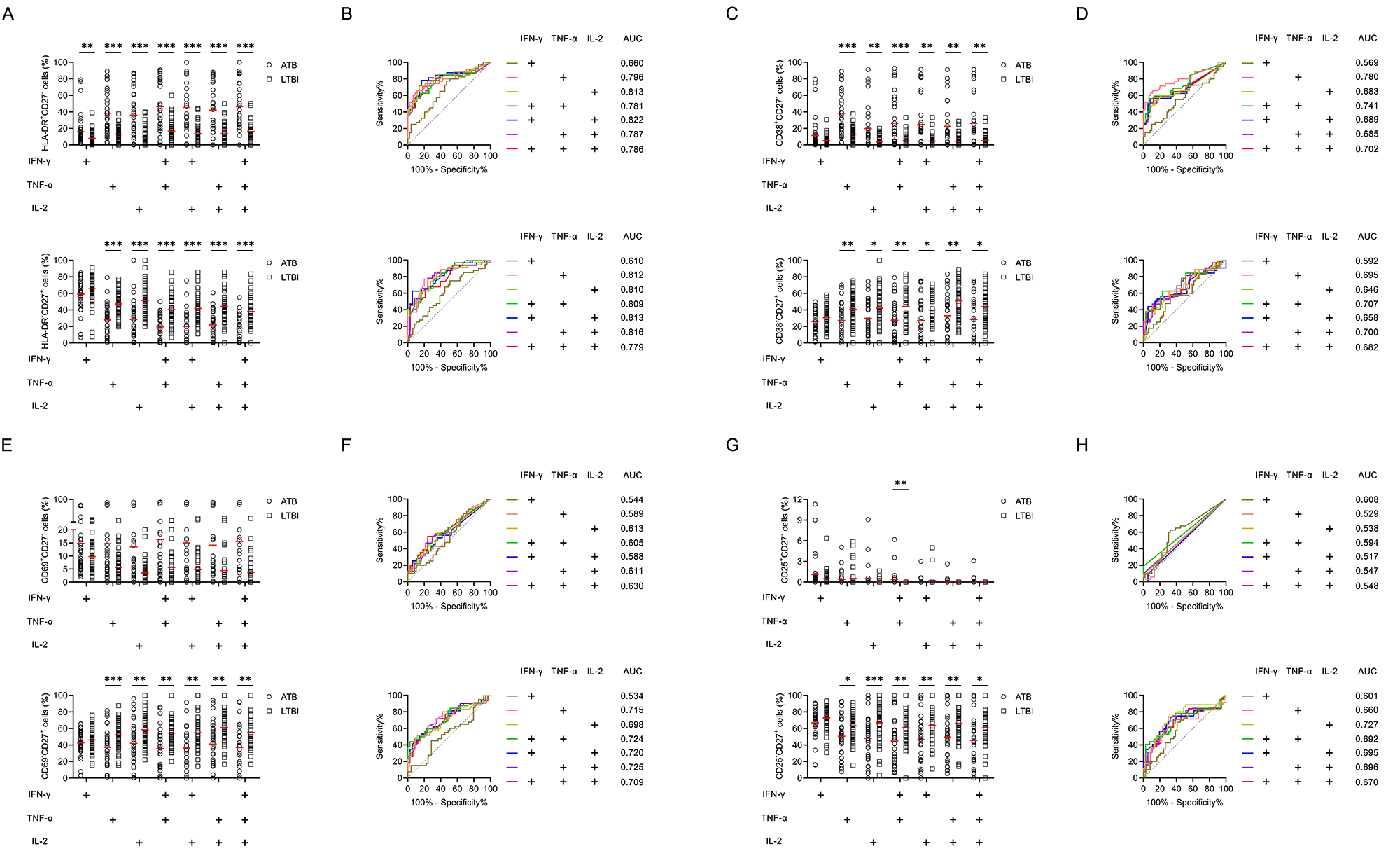

Supplement: Supplementary Figure 1 — The performance of the combination of activation markers and CD27 on MTB-specific cells in distinguishing ATB patients from LTBI individuals in Qiaokou cohort. (A) Aligned dot plots showing the proportions of HLA-DR+CD27- and HLA-DR-CD27+ cells of MTB-specific cells in ATB patients and LTBI individuals. Horizontal lines indicate the medians. (B) ROC analysis showing the performance of the proportions of HLA-DR+CD27- and HLA-DR-CD27+ cells of MTB-specific cells in discriminating ATB patients from LTBI individuals. (C) Aligned dot plots showing the proportions of CD38+CD27- and CD38-CD27+ cells of MTB-specific cells in ATB patients and LTBI individuals. Horizontal lines indicate the medians. (D) ROC analysis showing the performance of the proportions of CD38+CD27- and CD38-CD27+ cells of MTB-specific cells in discriminating ATB patients from LTBI individuals. (E) Aligned dot plots showing the proportions of CD69+CD27- and CD69-CD27+ cells of MTB-specific cells in ATB patients and LTBI individuals. Horizontal lines indicate the medians. (F) ROC analysis showing the performance of the proportions of CD69+CD27- and CD69-CD27+ cells of MTB-specific cells in discriminating ATB patients from LTBI individuals. (G) Aligned dot plots showing the proportions of CD25+CD27- and CD25-CD27+ cells of MTB-specific cells in ATB patients and LTBI individuals. Horizontal lines indicate the medians. (H) ROC analysis showing the performance of the proportions of CD25+CD27- and CD25-CD27+ cells of MTB-specific cells in discriminating ATB patients from LTBI individuals. *P < 0.05, **P < 0.01, ***P < 0.001 (Mann-Whitney U test). MTB, Mycobacterium tuberculosis; ATB, active tuberculosis; LTBI, latent tuberculosis infection; AUC, area under the curve. [file Image_1.tif]
