# Supplementary material for: Activation Phenotype of Mycobacterium tuberculosis-Specific CD4+ T Cells Promoting the Discrimination Between Active Tuberculosis and Latent Tuberculosis Infection
Source: Front Immunol. 2021 Aug 26;12:721013. doi: 10.3389/fimmu.2021.721013 (PMC8426432; doi:10.3389/fimmu.2021.721013)
Supplement: Supplementary file 2 [file Table_1.docx]

| **Supplementary Table 1. The antibodies used in this study** | | |
| --- | --- | --- |
| Antibodies | Manufacturer | Clone and cat |
| Anti-human CD4 APC-Cy7 | Biolegend | Clone:RPA-T4; Cat#300518 |
| Anti-human IFN-γ BV605 | Biolegend | Clone:4S.B3; Cat#502536 |
| Anti-human TNF-α FITC | Biolegend | Clone:MAb11; Cat#502906 |
| Anti-human IL-2 PE | Biolegend | Clone:MQ1-17H12; Cat#500307 |
| Anti-human CD25 APC | Biolegend | Clone:BC96; Cat#302610 |
| Anti-human CD69 BV421 | Biolegend | Clone:FN50; Cat#310930 |
| Anti-human HLA-DR PerCp 5.5 | Biolegend | Clone:L243; Cat#307630 |
| Anti-human CD38 BV510 | Biolegend | Clone:HIT2; Cat#303540 |
| Anti-human CD27 PE-Cy7 | Biolegend | Clone:O323; Cat#302838 |
